# Association between the functional brain network and antidepressant responsiveness in patients with major depressive disorders: a resting-state EEG study

**DOI:** 10.1017/S0033291724003477

**Published:** 2025-02-06

**Authors:** Kang-Min Choi, Hyeon-Ho Hwang, Chaeyeon Yang, Bori Jung, Chang-Hwan Im, Seung-Hwan Lee

**Affiliations:** 1 Clinical Emotion and Cognition Research Laboratory, Inje University, Goyang, Republic of Korea; 2 Department of Electronic Engineering, Hanyang University, Seoul, Republic of Korea; 3 Department of Human-Computer Interaction, Hanyang University, Ansan, Republic of Korea; 4 Department of Psychology, Sogang University, Seoul, Republic of Korea; 5 Department of Biomedical Engineering, Hanyang University, Seoul, Republic of Korea; 6 Department of Psychiatry, Ilsan Paik Hospital, Inje University College of Medicine, Goyang, Republic of Korea; 7 Bwave Inc, Juhwa-ro, Goyang, Republic of Korea

**Keywords:** antidepressant responsiveness, electroencephalography, functional brain network, graph theory, major depressive disorder, resting state

## Abstract

**Background:**

Recent neuroimaging studies have demonstrated that the heterogeneous antidepressant responsiveness in patients with major depressive disorder (MDD) is associated with diverse resting-state functional brain network (rsFBN) topology; however, only limited studies have explored the rsFBN using electroencephalography (EEG). In this study, we aimed to identify EEG-derived rsFBN-based biomarkers to predict pharmacotherapeutic responsiveness.

**Methods:**

The resting-state EEG signals were acquired for demography-matched three groups: 98 patients with treatment-refractory MDD (trMDD), 269 those with good-responding MDD (grMDD), and 131 healthy controls (HCs). The source-level rsFBN was constructed using 31 sources as nodes and beta-band power envelope correlation (PEC) as edges. The degree centrality (DC) and clustering coefficients (CCs) were calculated for various sparsity levels. Network-based statistic and one-way analysis of variance models were employed for comparing PECs and network indices, respectively. The multiple comparisons were controlled by the false discovery rate.

**Results:**

Patients with trMDD were characterized by the altered dorsal attention network and salience network. Specifically, they exhibited hypoconnection between eye fields and right parietal regions (*p* = 0.0088), decreased DC in the right supramarginal gyrus (*q* = 0.0057), and decreased CC in the reward circuit (*q*s < 0.05). On the other hand, both MDD groups shared increased DC but decreased CC in the posterior cingulate cortex.

**Conclusions:**

We confirmed that network topology was more severely deteriorated in patients with trMDD, particularly for the attention-regulatory networks. Our findings suggested that the altered rsFBN topologies could serve as potential pathologically interpretable biomarkers for predicting antidepressant responsiveness.

## Introduction

Major depressive disorder (MDD) is a heterogeneous mental disorder. This heterogeneity is attributable to a variety of factors, such as genotypic variations, pathophysiological symptoms, and etiological triggers (Al-Harbi, 2012; American Psychiatric Association, 2013; Fonzo et al., 2019; Jermy et al., 2021; Kennis et al., 2020), contributing to varying individually optimal therapeutic strategies. In fact, ~30% of patients with MDD do not well-respond to antidepressant treatment, one of the most widely used and validated therapies (Al-Harbi, 2012; Iseger et al., 2017; Wu et al., 2022). To date, numerous MDD studies have focused on the identification of biomarkers for predicting pharmacological treatment responsiveness to minimize unnecessary side effects and reduce treatment duration due to the suboptimal therapy in patients with treatment-resistant MDD (trMDD) (Al-Harbi, 2012; Iseger et al., 2017; Kennis et al., 2020; Wu et al., 2022).

Neuroimaging studies could potentially aid in identifying neurobiologically established biomarkers to predict pharmacotherapeutic responsiveness. Notably, the resting-state functional brain network (rsFBN) offers a promising approach to yield reliable and informative insights into mental illnesses. It covers broad spontaneous functional architecture without cognitively demanding tasks, facilitating multi-site investigations (Dichter et al., 2015; Rolle et al., 2020; Woodward & Cascio, 2015; Zhang et al., 2021). Among various neuroimaging modalities, functional magnetic resonance image (fMRI) has been predominantly employed to explore network topologies for investigating treatment-resisting traits, owing to its superior spatial resolution (Dichter et al., 2015; Woodward & Cascio, 2015). Nevertheless, the fMRI comes with several inherent limitations, including its high cost, demand for technical expertise, and specialized equipment (Rolle et al., 2020; Zhang et al., 2021).

Electroencephalography (EEG) could be a viable alternative modality for exploring rsFBNs in clinical environments because it captures direct electrophysiological brain activity with less cost and environmental requirements (Hipp et al., 2012; Rolle et al., 2020; Zhang et al., 2021). Recently, multimodal neuroimaging studies have demonstrated that EEG-based rsFBNs employing high-frequency power-envelope correlation (PEC) as an functional connectivity (FC) metric display overlapping patterns with fMRI-based rsFBNs (Brookes et al., 2011; Hipp et al., 2012; Siems et al., 2016; Zhang et al., 2021). These findings support the reliability of the pathophysiological findings derived from EEG-based rsFBNs, reinforcing neurobiological mechanisms previously established through fMRI research.

Several PEC-based rsFBN studies have found pathophysiological characteristics of the treatment-resistant profiles. For example, (Zhang et al., 2021) identified that those who exhibited relatively intact rsFBNs tended to sensitively respond to pharmacotherapy, particularly for beta bands showing overlapping patterns with fMRI. Rolle et al. (2020) found trMDD-specific rsFBN alterations, which were associated with anhedonia symptoms. While these studies have demonstrated the feasibility of using the PEC-driven rsFBN to identify biomarkers with relatively large sample sizes, further replication is still needed to generalize and refine these findings across various cohorts. Moreover, complex network analysis has been scarcely explored so far. The graph theory-based higher-order topological analysis could provide insightful comprehensive network characteristics (Rubinov & Sporns, 2010), potentially linked to the treatment-resisting pathophysiological characteristics.

In the present study, we explored the predictors for antidepressant responsiveness in patients with MDD using EEG-based rsFBN patterns. Specifically, various PEC-driven network topological characteristics, including complex network indices, were compared for three groups with sufficient sample sizes: (i) treatment-resisting MDD (trMDD), (ii) good-responding MDD (grMDD), and (iii) healthy control (HC). Based on the comparative analysis, we sought to identify the association between functional connectome-wise neurobiological aberration and treatment-resisting pathophysiological traits. In line with previous studies, it is hypothesized that trMDD would exhibit more severe rsFBN alteration compared to grMDD.

## Methods and materials

### Participants

A total of 553 patients with MDD were included in this retrospective study at the Inje University Ilsan Paik Hospital from October 2010 to March 2021. All the patients were diagnosed by certified psychiatrists using the criteria of the Diagnostic and Statistical Manual of Mental Disorders, 4th edition, text revision (DSM-IV-TR) or 5th edition (DSM-V). Among them, several patients were excluded from further analysis based on the following criteria: (i) organic brain damage or neurological disorder, (ii) comorbidity with schizophrenia, bipolar disorder, post-traumatic stress disorder, and obsessive-compulsive disorder, (iii) primary diagnosis with substance abuse, (iv) intellectual disability, (v) pregnancy, (vi) comorbidity with psychotic symptoms, (vii) atypical depression, and (viii) too mild depressive episode (Supplementary material). Several patients were also excluded due to the poor data quality (Supplementary material). Finally, eligible 367 patients were divided into two subgroups: trMDD (*n* = 98, aged 42.47 ± 12.87, Male: 21.43%) and grMDD (*n* = 269, aged 42.26 ± 12.85, Male: 20.07%).

The trMDD was determined when patients showed a lack of improvement after 8 weeks of treatment with two or more antidepressants. Among the patients with MDD, trMDD was defined as having at least one of the following criteria: (i) those who need to be treated with the mood stabilizer, aripiprazole, quetiapine, or other antipsychotic medications (Carvalho et al., 2009; Fava, 2003), (ii) who suffer from severe levels of depressive episode with suicidal ideation (Bergfeld et al., 2018; Sun et al., 2016), and (iii) who were comorbid with panic disorder, personality disorder, eating disorder, attention deficit hyperactivity disorder, or substance disorder (Brenner et al., 2020; Kratochvil et al., 2009; Thase, 1996). The determination was made by certified psychiatrists after at least two types of antidepressant therapy, the duration of which lasted for over 8 weeks. The other patients who did not satisfy the above criteria of treatment refractoriness were regarded as grMDD. It is noted that patients who showed medication compliance deficit or whose dosage is restricted due to several reasons were categorized as pseudo-resistant MDD, who were excluded in our study. Their baseline depressive symptom severity was assessed based on the Patient Health Questionnaire-9 (PHQ-9) (Kroenke et al., 2001) score and the International Classification of Diseases (ICD) code, acquired by a physician’s interview (Supplementary material).

Moreover, a total of 157 HCs were recruited from the local community. None of them had suffered from any major psychiatric, neurologic disorders, head injury, or had a family history of psychiatric disorders. Several participants were excluded due to the data quality issue. As a result, the analysis was conducted with 131 HCs (aged 42.97 ± 14.50, Male: 27.48%). All the participants provided their written informed consent.

This study was approved by the Inje University Ilsan Paik Hospital Institutional Review Board (IRB no. 2018-12-012-013). The requirement for written informed consent was waived by the IRB due to the retrospective nature of our study. The data collection for HCs was also conducted following the ethical guidelines (IRB no. 2015-07-026).

### Signal acquisition and pre-processing

A total of 64 electrodes were placed following the extended 10–20 system EEG signal recording. In addition, four electrodes and one electrode were placed to acquire electrooculogram and electrocardiogram signals, respectively. The recordings were conducted under eyes-closed conditions. The signals were recorded at 1,000 Hz of sampling rate with band-pass filtered between 0.1 and 100 Hz.

The signal preprocessing was performed using the EEGLAB toolbox (Delorme & Makeig, 2004) implemented in MATLAB R2019b (MathWorks, Natick, MA, USA). The artifactual components were manually rejected using an independent component analysis algorithm. The EEG signals were band-pass filtered between 1 and 50 Hz and then common-average referenced. The cleaned EEG signals were segmented into 2 s without any overlaps. For more details, see the Supplementary material.

### Construction of functional network

Source signals were computed using the Brainstorm toolbox (Tadel et al., 2011). Thirty-one regions of interest (ROIs) were selected as the representative nodes of the functional network according to the previous fMRI studies (Rolle et al., 2020; Toll et al., 2020; Zhang et al., 2021). The ROIs were incorporated into one of the six modules (Table S1 and Figure S1): (i) visual network (VN; R1 ~ 2), (ii) somatosensory network (SMN; R3 ~ 4), (iii) dorsal attention network (DAN; R5 ~ 12), (iv) default mode network (DMN; R13 ~ 16), (v) central executive network (CEN; R17 ~ 24), and (vi) salience network (SN; R25 ~ 31). The orthogonalized power-envelope correlation (PEC) was evaluated between a pair of nodes for the beta band (12–30 Hz) as edges between the nodes (details in Supplementary material).

### Network indices and minimum-spanning tree

Prior to the calculation of the complex network indices, the rsFBNs were binarized using a data-driven multiple thresholding approach, across 5 to 35% of the FC proportions with an interval of 5% to avoid potential bias due to the specific threshold value (Figs. S2 and S3) (Rubinov & Sporns, 2010; Zhang et al., 2018). The representative network indices were determined as the averaged values across the multiple thresholds. To investigate higher-order network topological characteristics, several network indices were calculated using the brain connectivity toolbox (Rubinov & Sporns, 2010): degree centrality (DC), clustering coefficients (CC), and efficiency (Eff). The representative minimum-spanning tree (MST) was also calculated to effectively visualize the connectome characteristics for each group.

### Statistical analysis

Because the absolute skewness and kurtosis values of all the data distribution were <2 and 7, respectively, all the data distribution was assumed to be normal (Curran et al., 1996). To test the demographic differences between the three groups (i.e., trMDD, grMDD, and HC), one-way analysis of variance (ANOVA) and the Chi-squared test were employed for the age and sex, respectively. The pre-treatment symptom severity between trMDD and grMDD was compared using a Chi-squared test.

The FCs were compared using the network-based statistic (NBS) toolbox, which reveals significantly different subnetworks with the family-wise error rate effectively controlled (Zalesky et al., 2010). Specifically, the ANOVA model was employed to compare three groups, and then the t-test model was employed for post hoc analysis. In the post hoc analysis, the alpha level was adjusted to 0.0167 (0.05/3) to assess multiple comparison issues. All the other parameters were set as default values.

The network indices were compared using the ANOVA model for three groups. For post hoc analysis, an independent t-test was employed. It is noted that the values exceeding three times the standard deviation from the distributions were determined as outliers and then discarded from the group comparison. The statistical test was performed using MATLAB and SPSS software (SPSS, Inc., Chicago, IL, USA). To address the multiple comparison issues, *p*-values were adjusted using false discovery rate (FDR) correction; the corrected *p*-values were then referred to as *q*-values.

## Results

### Demographical and clinical measures

There were no significant demographical and clinical differences among the groups (Table 1).

**Table 1.** Demographic information and depressive symptom severity for groups

	trMDD (*n* = 98)	grMDD (*n* = 269)	HC (*n* = 131)	p
Age	42.47 ± 12.87	42.26 ± 12.85	42.97 ± 14.50	0.8811
Sex (male/female)	21/77	54/215	36/95	0.2414
Symptom severity (moderate/severe)	48/50	134/135		0.9065

The symptom severity was assessed and recorded by the International Classification Diseases (ICD) code (details in Supplementary material): (i) moderate depressive symptom (F32.1) and (ii) severe depressive symptom without psychotic symptom (F32.2).

### Functional connectivity

**Figure 1.**
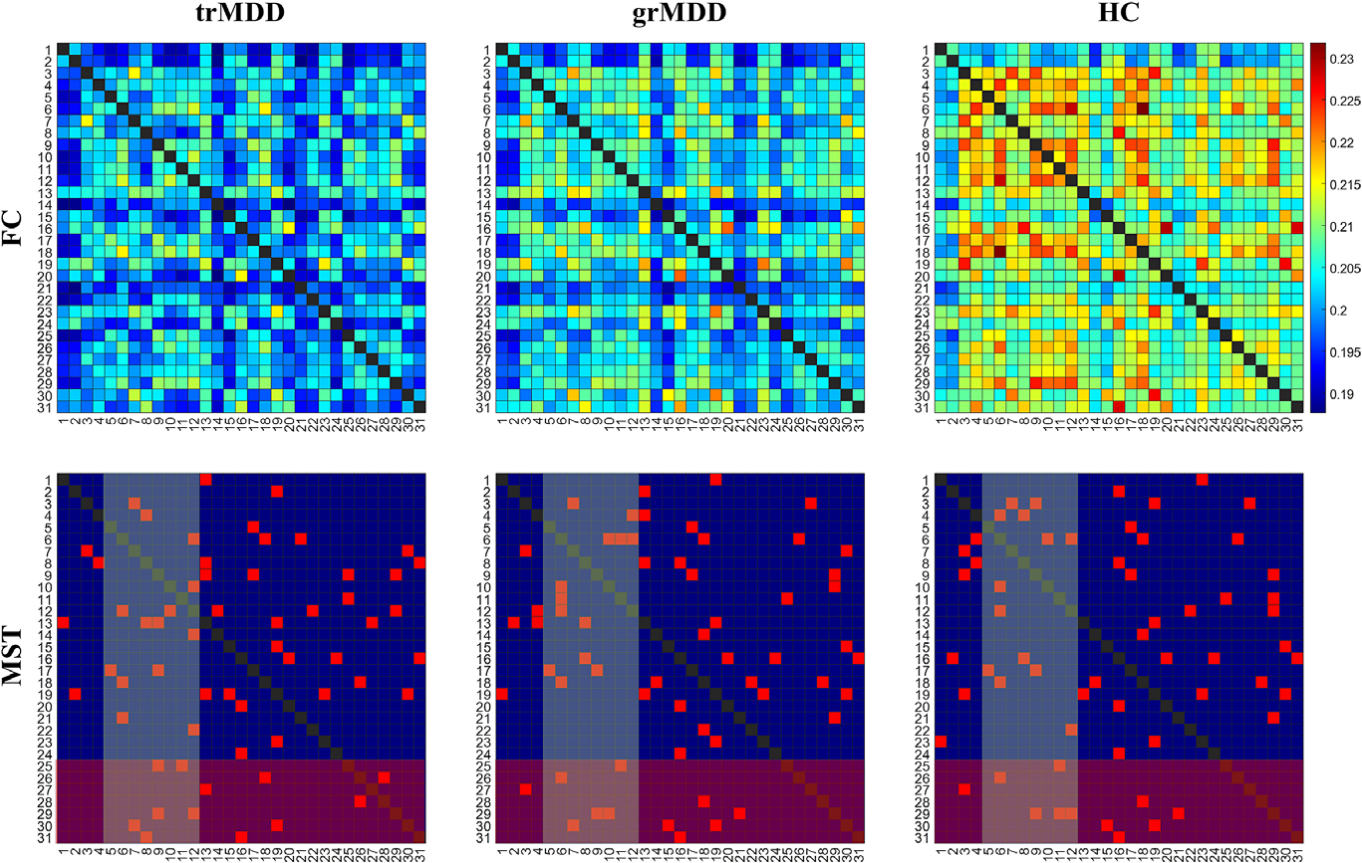
Comparison of the network connectome for each group. The upper and lower panels display grand averaged FCs for each node and their backbone connectome calculated by the MST algorithm, respectively. For the lower panels, the DAN (R5 to R12) and SN (R25 to R31)-related connectomes are highlighted with yellow and red boxes, respectively. The indices of the nodes correspond to the Table S1. DAN, dorsal attention network; SN, salience network; FC, functional connectivity; MST, minimum-spanning tree; trMDD, treatment-refractory MDD; grMDD, good-responding MDD; HC, healthy control.

Qualitatively, both MDD groups exhibited hypoconnectivity compared to HCs, with trMDD showing more severe alterations (Figure 1). The MST results also displayed that the connectome for trMDD was substantially altered compared to that for grMDD, particularly within the DAN and SN.

**Figure 2.**
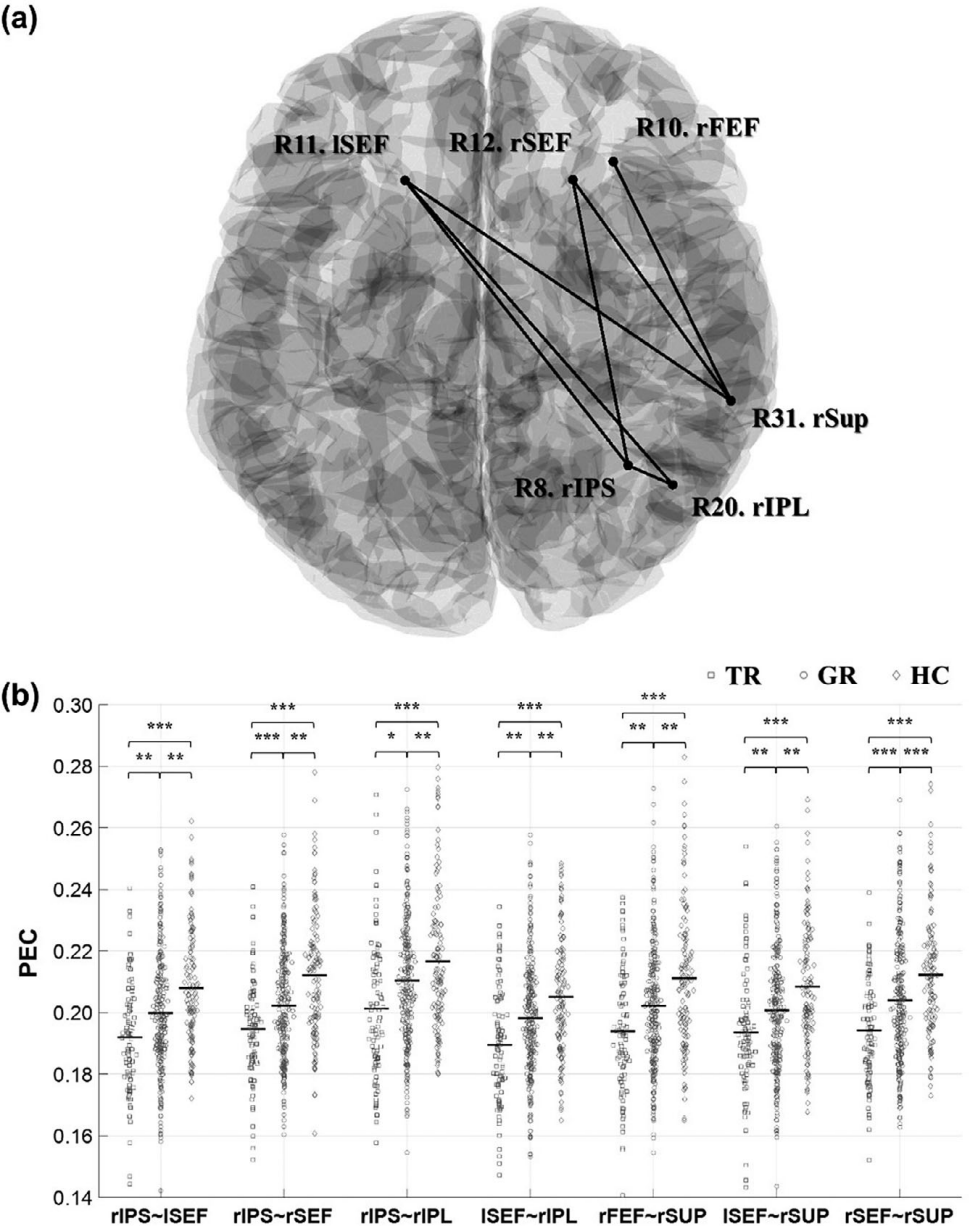
Hypoconnected FCs for trMDD compared to grMDD. (a) A subnetwork identified by NBS. The subnetwork consists of six nodes and seven edges. The under-connection was predominant for the DAN (R5 to R12), particularly for the frontal and supplementary eye fields and multi-modular right hemispheric parietal cortex. The dots and solid lines indicate the position of the nodes and significantly different FCs. The brain template image was acquired from the Brainstorm toolbox. (b) Group comparisons of the FCs. For each FC, trMDD (square), grMDD (circle), and HC (diamond) data are sequentially presented. Each dot represents the individual FC value, with their grand average values indicated with the central solid lines. TR, trMDD; GR, grMDD; l-, left; r-, right; IPS, intraparietal sulcus; SEF, supplementary eye field; FEF, frontal eye field; IPL, inferior parietal lobule; Sup, supramarginal gyrus. *q < 0.05; **q < 0.01; ***q < 0.001.

NBS results showed broad differences in FCs across groups (*n*_FC_ = 410, *p* < 0.0001, Figure S4). Post hoc analyses revealed a significant decrease in a variety of FCs for trMDD (*n*_FC_ = 291, *p* < 0.0001) and grMDD (*n*_FC_ = 248, *p* < 0.0001) compared to HC. NBS also revealed a significantly hypo-connected subnetwork for the trMDD compared to grMDD (*n*_FC_ = 7, *p* = 0.0088, Figure 2), specifically between the frontal and supplementary eye fields and right hemispheric parietal regions. The subnetwork primarily consisted of nodes in the DAN (R5 to R12), including the right inferior parietal sulcus (R8. rIPS), right frontal eye field (R10. rFEF), and both left and right supplementary eye fields (R11. lSEF and R12. rSEF). Additionally, the subnetwork included an SN node (right supramarginal gyrus, R31. rSup) and a CEN node (right inferior parietal lobule, R20. rIPL).

### Network indices

#### Degree centrality

**Figure 3.**
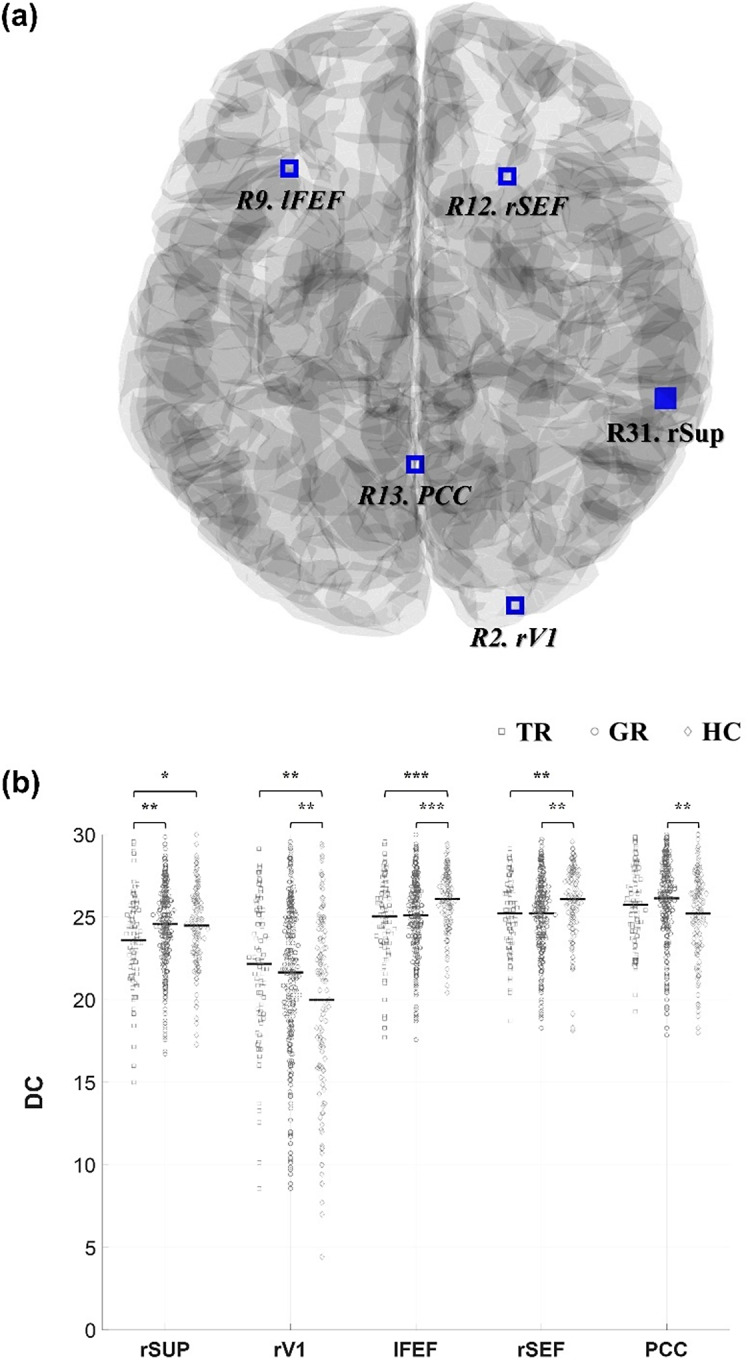
Group comparison for DCs. (a) Cortical regions showing significantly different DCs. Among them, a region showing a significant difference between trMDD and grMDD (i.e., rSup) is highlighted with filled blue; the others are marked with italics. The brain template image was acquired from the Brainstorm toolbox. (b) Group comparisons of the DCs. For each DC, trMDD (square), grMDD (circle), and HC (diamond) data are sequentially presented. Each dot represents the individual DC value, with their grand average values indicated with the central solid lines. DC, degree centrality; TR, trMDD; GR, grMDD; l-, left; r-, right; FEF, frontal eye field; SEF, supplementary eye field; PCC, posterior cingulate cortex; Sup, supramarginal gyrus; V1, visual cortex. *q < 0.05; **q < 0.01.

Among 31 DCs, five of them showed significant group differences (Figure 3): right visual cortex (R2. rV1, *q* = 0.0156), left FEF (R9. lFEF, *q* = 0.0039), rSEF (R10, *q* = 0.0187), posterior cingulate cortex (R13. PCC, *q* = 0.0232), and rSup (R31, *q* = 0.0350). Post hoc analyses revealed various group-dependent characteristics. First, trMDD showed decreased DC for the rSup compared to grMDD (trMDD = 23.61 versus grMDD = 24.58; *q* = 0.0057) and HC (versus HC = 24.50, *q* = 0.0205). Second, both trMDD and grMDD showed decreased DC for the lFEF (trMDD = 25.04, grMDD = 25.10 versus HC = 26.11; *q*s = 0.0004 and 0.0001, respectively) and rSEF (25.23, 25.22 versus 26.09; *q*s = 0.0066 and 0.0023). On the other hand, both trMDD and grMDD showed increased DCs for the rV1 (trMDD = 22.17, grMDD = 21.65 versus HC = 19.98; both *q*s = 0.0028, respectively). Although both trMDD and grMDD showed increased DC for the PCC compared to HC, only grMDD reached the significant level (25.74, 26.14 versus 25.21; *q*s = 0.1768 and 0.0024). There were no other significant group differences for the DCs.

#### Clustering coefficient

**Figure 4.**
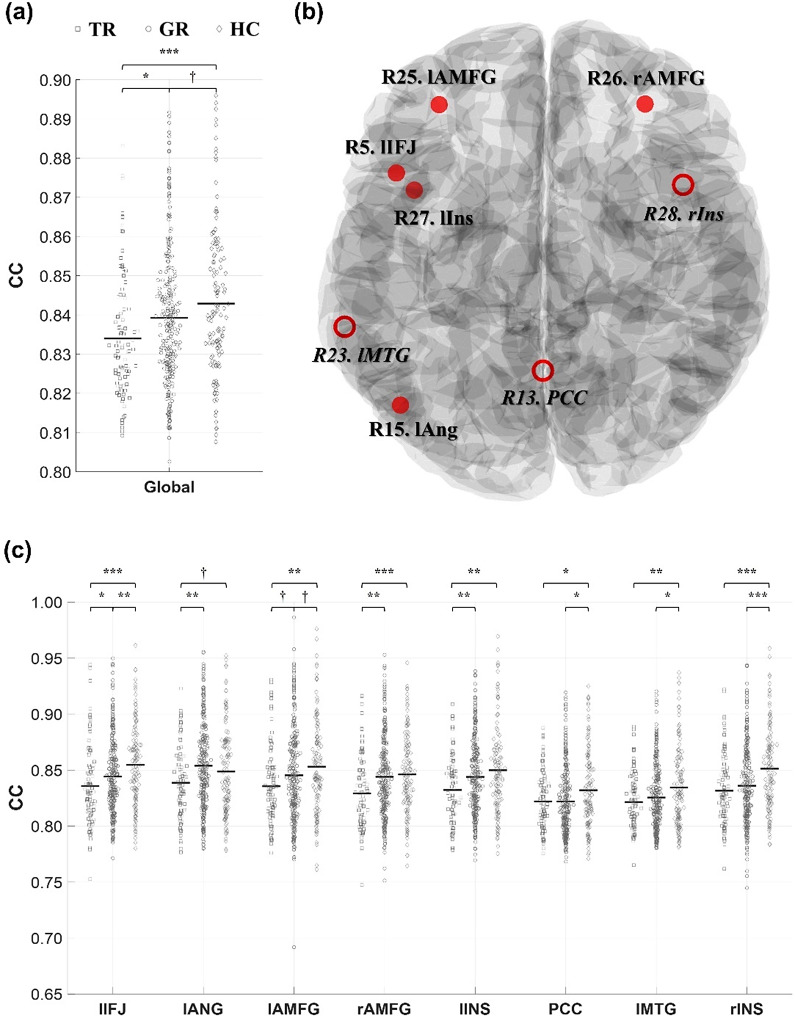
Group comparison for CCs. (a) Group comparison of the global CC. (b) Cortical regions showing significantly different CCs. Among them, regions showing significant differences between trMDD and grMDD (i.e., lAMFG, rAMFG, lIFJ, lIns, and lAng) are highlighted with filled red; the others are marked with italics. The brain template image was acquired from the Brainstorm toolbox. (c) Group comparisons of the CCs. For each CC, trMDD (square), grMDD (circle), and HC (diamond) data are sequentially presented. Each dot represents the individual CC value, with their grand average values indicated with the central solid lines. CC, clustering coefficient; TR, trMDD; GR, grMDD; l-, left; r-, right; AMFG, anterior middle frontal gyrus; IFJ, inferior frontal junction; Ins, insula; MTG, middle temporal gyrus; PCC, posterior cingulate cortex; Ang, angular gyrus. ^†^*q* < 0.09; *q < 0.05; **q < 0.01; ***q < 0.001.

There was a significant group difference in the global CC (*q* = 0.0010, Figure 4). Post hoc analyses revealed that trMDD exhibited under-clustering compared to grMDD (trMDD = 0.8340 versus grMDD = 0.8393; *q* = 0.0153) as well as HC (versus HC = 0.8429; *q* = 0.0007). Moreover, grMDD also exhibited a decreased trend of global CC (*q* = 0.0650).

A similar tendency was observed in the nodal CCs (i.e., trMDD < grMDD < HC), particularly for the SN nodes (R25 to R31). Specifically, group differences were observed in the left inferior frontal junction (R5. lIFJ, *q* = 0.0055), posterior cingulate cortex (R13. PCC, *q* = 0.0301), left angular gyrus (R15. lAng, *q* = 0.0212), left middle temporal gyrus (R24. lMTG, *q* = 0.0212), left and right anterior mid-frontal gyrus (R25. lAMFG, *q* = 0.0301; R26. rAMFG, *q* = 0.0075), and left and right insula (R27. lIns, *q* = 0.0075; R28. rIns, *q* = 0.0006). Post hoc analyses revealed that trMDD showed decreased nodal CCs compared to grMDD as well as HC in the lIFJ (trMDD = 0.8358 versus grMDD = 0.8444, *q* = 0.0415; versus HC = 0.8547, *q* = 0.0005), lAng (0.8387 versus 0.8539, *q* = 0.0025; versus 0.8487, *q* = 0.0716), lAMFG (0.8356 versus 0.8453, *q* = 0.0674; versus 0.8531, *q* = 0.0028), rAMFG (0.8291 versus 0.8440, *q* = 0.0010; versus 0.8462, *q* = 0.0010), and lIns (0.8323 versus 0.8439, *q* = 0.0052; versus 0.8499, *q* = 0.0011); among them, grMDD also showed decreased nodal CCs compared to HC in the lIFJ (*q* = 0.0098) and lAMFG (*q* = 0.0897).

For the other nodal CCs, both the trMDD and grMDD showed decreased values compared to HC without any significant differences between the MDD subgroups (*q*s > 0.1) in the PCC (trMDD = 0.8220, grMDD = 0.8220 versus HC = 0.8321; *q*s = 0.0280, and 0.0121, respectively), lMTG (0.8215, 0.8255 versus 0.8345; *q*s = 0.0088, 0.0147), and rIns (0.8316, 0.8361 versus 0.8512; *q*s = 0.0001, 0.0001). There were no other significant group differences for the CCs.

#### Efficiency

The global Eff was not compared because most of them showed equal values (0.9002, *n* = 400/498) mainly due to the same density (Figure S5). In addition, nodal Effs were also not compared due to an excessively high correlation to the nodal CCs (Pearson correlation, *r* = 0.9308), showing almost equivalent comparative results (Figure S6).

## Discussion

In this study, we delved into predictors for antidepressant treatment responsiveness using EEG-based rsFBN by comparing the network topologies of three demographically matched groups: trMDD, grMDD, and HC. Notably, while both MDD subgroups manifested shared topological alterations to some extent, the trMDD exhibited more pronounced aberrations as expected, mainly for two attentional regulation networks: DAN and SN. Specifically, trMDD was characterized by the hypoconnectivity between the eye fields and right parietal regions, predominantly for the DAN. The trMDD was also characterized by under-clustering, predominantly in the SN. Concurrently, a commonality between the MDD subgroups was the presence of altered neural circuits, indicative of internally-biased attentional flows.

In the current research, the orthogonalized beta-band PEC was employed to evaluate FC, the pattern of which was found to be similar to the fMRI-based FC (Brookes et al., 2011; Hipp et al., 2012; Siems et al., 2016; Zhang et al., 2021). Such congruency bolsters the reliability of interpreting EEG-derived rsFBN topologies in the context of pathophysiological studies that utilize fMRI as a neuroimaging modality. Among high-frequency bands, given that gamma-band EEG is susceptible to muscular artifacts and typically less active in the resting states, beta-bands would be better preferable for constructing reliable rsFBNs (Hipp et al., 2012; Siems et al., 2016). For the network binarization, a multiple proportion-based thresholding approach was employed to enhance reliability. Despite the prevailing view that thresholding is crucial in eliminating spurious connections to enhance pattern clarity, there is no gold standard so far (Hatlestad-Hall et al., 2021; Zhang et al., 2018). Nevertheless, our data-driven multiple proportional thresholding approach might yield a relatively more optimal range to differentiate trMDD.

Overall, alterations in the rsFBN were more severe in patients with trMDD compared to grMDD, consistent with previous neuroimaging studies (Fonzo et al., 2019; Martens et al., 2022; Zhang et al., 2021). These alterations might be associated with the distinct clinical symptoms of trMDD, rather than depressive symptom severity. Interestingly, the network alterations were predominant for two key attentional regulatory network modules: DAN and SN. The primary role of the DAN is voluntary top-down attentional control according to the intention and goals, the dysfunction of which is one of the major hallmarks in patients with MDD (Gao et al., 2023; Sternat et al., 2018). On the other hand, the SN primarily governs bottom-up attentional responses to external stimuli and affective control, the dysfunction of which is also commonly observed (Heerlein et al., 2021; Jaffe et al., 2019; Kaiser et al., 2015). Thus, our results support the attention deficit model in patients with trMDD (Sternat et al., 2018).

Patients with trMDD exhibited hypoconnection predominantly for the DAN. Specifically, the hypoconnection was mainly observed for the frontal and supplementary eye fields (i.e., rFEF and bilateral SEFs) and right-hemispheric parietal areas for multiple network modules. Among these cortical areas, it is remarkable that trMDD exhibited decreased DC only in the rSup (Figure 3). Given that trMDD showed relatively lower FCs between the rSup (in SN) and the eye fields (in DAN), it is comprehensively inferable that the rSup is a main information processing region, by interacting with the eye fields, including the rSEF. However, the rSup appears to lose its status as a hub in trMDD. This cortical region is involved in empathic judgment and mitigation of egocentric bias (Silani et al., 2013), which were compromised in trMDD (Kilian et al., 2022). On the other hand, both MDD groups shared decreased DCs for the eye fields; alternatively, those for rV1 increased. These findings are consistent with previous psychopathological studies reporting that patients with MDD exhibited aberrant visual attentional bias, even those who already have remitted (von Koch et al., 2023).

The nodal CCs were decreased in patients with trMDD, particularly for the SN nodes. Remarkably, the bilateral AMFGs and lIns are known to be associated with reward processing (Geugies et al., 2022; Rolle et al., 2022). The AMFG is the anterior part of the dorsolateral-prefrontal cortex (Fonzo et al., 2017), a central cortical region within the reward circuit, malfunctioning of which is linked to the disrupted affective controls and cognitive functions in MDD (Rolle et al., 2022). The malfunctioning reward circuit could be associated with dopaminergic dysregulation, leading to anhedonia symptoms (Szczypinski & Gola, 2018), which have been consistently found in treatment-resistant patients (Rolle et al., 2020; Sternat et al., 2018; Szczypinski & Gola, 2018). Moreover, the other nodes showing under-clustering for trMDD, including lIns, lIFJ, and lAng, also contribute to the reward processing (Geugies et al., 2022; Hippmann et al., 2019; Nimarko et al., 2022), further underpinning the anhedonia model.

Contrastingly, both MDD groups shared decreased nodal CCs for PCC, lMTG, and rIns. Notably, only the PCC showed overlapping alterations in both the CC and DC measures, despite their differing topological characteristics. Comprehensively, it is inferred that the reduced nodal clustering in the PCC potentially stems from its increased connections (i.e., increased denominator). It is consequently suggested that patients with MDD show abnormal increases in PCC-centered connections that are not reciprocally interconnected. The PCC is a crucial hub for DMN, hyperactivation of which is one of the common features of MDD (Dichter et al., 2015; Martens et al., 2022). This finding is associated with the imbalance between the internal (DMN) and external (CEN) attentional direction in the patients (Kaiser et al., 2015), potentially due to malfunctioning switching module (SN). This suggestion is supported by the decreased segregation in the rIns (in SN) and lMTG (CEN) for both MDD groups. Importantly, the rIns is a key node for switching the attentional direction (Sridharan et al., 2008). The inefficient information processing in the rIns might be associated with volumetric reduction in its gray matter in the early stage of the illness (Lu et al., 2023). Thus, these findings might be pathophysiologically reliable indicators for discriminating MDD from HC.

The current study has several limitations. First, the pharmacological effects could not be controlled. However, this effect on the resting-state FC is known to be trivial (Zhang et al., 2021). Second, the placebo effect was not considered. Third, our results should be carefully interpreted due to the limited cohort (Koreans exclusively). Finally, the static assumption on the rsFBN might oversimplify the actual neurophysiological model because the brain dynamically changes its functional states even in the resting state.

Our study demonstrated that trMDD exhibited pronounced rsFBN alterations compared to grMDD, which could serve as potential biomarkers. Specifically, our results support the previous clinical findings that trMDD exhibited attentional malfunctioning and anhedonia symptoms. Future research should focus on generalization to identify robust biomarkers predicting antidepressant responsiveness in patients with MDD.

## Supporting information

Choi et al. supplementary material Choi et al. supplementary material

## Supplementary material

The supplementary material for this article can be found at http://doi.org/10.1017/S0033291724003477.
