# Supplementary material for: Association between the functional brain network and antidepressant responsiveness in patients with major depressive disorders: a resting-state EEG study
Source: Psychol Med. 2025 Feb 6;55:e25. doi: 10.1017/S0033291724003477 (PMC12017359; doi:10.1017/S0033291724003477)
Supplement: Choi et al. supplementary material [file S0033291724003477sup001.docx]

**Supplementary material**

Contents

1. Methods
2. Details about clinical information
3. Details about signal acquisition and pre-processing
4. Details about constructing functional network
5. Determination of threshold for network binarization
6. Results
7. Group comparison for the numbers of epochs
8. Optimal proportional threshold interval
9. NBS results
10. Efficiency
11. Network indices for the random networks

**1. Supplementary methods**

1.1. Details about clinical information

The depressive symptom severity was assessed based on the PHQ-9 score and ICD code. For the PHQ-9, symptom severity was divided into mild (5 to 9), moderate (10 to 14), and severe (> 15) (Kroenke et al. 2001). The diagnosis of depression was documented in the medical records according to the ICD coding system, in accordance with South Korea's medical law [1]. All patients were included one of the (i) ‘F32.0: mild depressive episode,’ (ii) ‘F32.1: moderate depressive episode,’ (iii) ‘F32.2: severe depressive episode without psychotic symptoms,’ (iv) ‘F32.3: severe depressive episode with psychotic symptoms,’ (v) ‘F32.8: atypical depression.’ As mentioned in the main text, those exhibited too mild depressive episode (i.e., F32.0) were excluded in the further analysis due to possible sample imbalance (trMDD = 0 vs. grMDD = 19). In addition, those with psychotic symptoms (i.e., F32.3; trMDD = 4 vs. grMDD = 3) and atypical depression (trMDD = 2 vs. grMDD = 5) were also excluded due to the limited samples.

1.2. Details about signal acquisition and pre-processing

The participants were seated in a comfortable chair with their eyes closed. Biosignals were acquired using Neuroscan SynAmps2 (Compumedics USA, El Paso, TX, USA). A total of 64 Ag-AgCl electrodes mounted on a Quik-Cap were placed following the extended 10-20 system to record EEG signals. Four additional electrodes were placed on the outer canthi and above and below the left eye to acquire electrooculogram (EOG) signals. One additional channel was attached to acquire electrocardiogram (ECG) signals. During the signal acquisition, the impedance of all the electrodes was carefully maintained below 5 kΩ. The signals were recorded at 1,000 Hz of sampling rate with band-pass filtered between 0.1 – 100 Hz. Several patients conducted multiple trials of EEG recordings; in that cases, initially acquired EEG signals were used for the data analyses.

The independent component analysis (ICA) was applied for 65 channels of biosignals, consisting of 62 channels for EEG, two bipolar channels for EOG (i.e., vertical and horizontal), and one channel for ECG. At most 20% of independent components (ICs) were determined as artifacts, including EOG, ECG, and EMG, and then eliminated (i.e., a maximum of 13 ICs). The non-EEG channels were then discarded in the further steps. The bad channels were interpolated at most 5% (i.e., 3 channels). Afterwards, bad blocks were manually rejected. The EEG signals were band-pass filtered between 1 – 50 Hz using a 6^th^-order Butterworth filter and then common-average referenced. The cleaned EEG signals were segmented into 2 s without any overlaps. All the epochs whose absolute maximum values exceeded 75 μV were excluded from the analysis. In these steps, data contaminated with excessive artifacts or bad channels were excluded in the further analysis. In addition, participants with the number of epochs less than 45 were also excluded [2].

1.3. Details about constructing functional network

The source activities were reconstructed using a Brainstorm toolbox [3]. In detail, depth-weighted L2-norm estimator from the preprocessed scalp EEG signals. A total of 15,002 voxels originating from the Colin 27 MRI brain template were employed for source reconstruction. A three-layer boundary element model implemented from the OpenMEEG project software was utilized for constructing the lead field matrix [4].

For constructing the rsFBN, we employed a resting-state fMRI-based template [5]. In the template, there are a total of 31 ROIs belonging to six network modules (Table S1, Figure S1). For each ROI, voxels within a 5 mm Euclidean distance from its representative coordinate were used for computing source activity. Finally, the representative signals of each ROI were reconstructed as the first component computed by the principal component analysis from these neighboring voxels. Please refer to the study by Rolle et al. [6] for the Montreal Neurologic Institute (MNI) coordinates.

For each 2 s epoch, a pair of the representative source signals of the ROIs were band-pass filtered according to the beta band using a 6th-order Butterworth filter. Subsequently, the analytic signals were calculated using a Hilbert transform. To mitigate the volume conduction effect, orthogonalization was applied to the complex signals [7]. Finally, the PECs were evaluated as the absolute correlation coefficients of the log-transformed instantaneous powers. To mitigate the edge effect caused by the filtering and Hilbert transform, 100 ms from both ends of the segments were discarded from the evaluation. It is noted that the PEC values can vary from 0 (out-of-phase) to 1 (total phase-locked). The detailed information for calculating PEC is delineated in the study of Toll et al [8].

As network measures, DCs, global and nodal CCs, and Effs were calculated. Specifically, DC was evaluated as the number of connections that the node possesses. It should be noted that the DCs were computed and compared solely for the nodal level, as our binarization approach inherently resulted in identical global-level DCs. The global and nodal CCs were calculated to assess network segregation. Nodal CC represents the probability that two arbitrary nodes are connected, given that they both share a common ‘basis’ node; the global CC is the average of all nodal CC values within the network. Global Eff was evaluated to assess the functional integration of the network, which is evaluated by the averaged inverse shortest path length. Nodal Eff was evaluated to assess the functional integration of the subnetwork consisting of the nodes which are connected to the ‘basis’ one, which ultimately represents the network segregation and resiliency. The overall indices were calculated using the brain connectivity toolbox [8].

The backbones of the networks were visualized using the minimum spanning tree (MST) algorithm to compare network connectomes qualitatively. The MST yields circuits consisting of the minimum FCs spanning all of the nodes without any triangles. It was also calculated using the brain connectivity toolbox [9], based on the grand averaged rsFBNs for each group.

**Table S1**. Detailed information about the ROIs. The labels of the modules and ROIs are presented with their indices left and right sides, respectively.

| Module | ROI labels | |
| --- | --- | --- |
| 1. VN | 1) lV1, 2) rV1 |  |
| 2. SMN | 3) lSMC, 4) rSMC |  |
| 3. DAN | 5) lIFJ, 6) rIFJ, 7) lIPS, 8) rIPS, 9) lFEF, 10) rFEF, 11) lSEF, 12) rSEF |  |
| 4. DMN | 13) PCC, 14) mPFC, 15) lAng, 16) rAng |  |
| 5. CEN | 17) lPMFG, 18) rPMFG, 19) lIPL, 20) rIPL, 21) lOrb, 22) rOrb, 23) lMTG, 24) rMTG |  |
| 6. SN | 25) lAMFG, 26) rAMFG, 27) lIns, 28) rIns, 29) dACC, 30) lSup, 31) rSup |  |

VN, visual network; SMN, somatosensory network; DAN, dorsal attention network; DMN, default mode network; CEN, central executive network (or frontoparietal network); SN, salience network (or ventral attention network); l-, left hemispheric; r-, right hemispheric; V1, visual cortex; SMC, somatosensory cortex; IFJ, inferior frontal junction; IPS, intraparietal sulcus; FEF, frontal eye field; SEF, supplementary eye field; PCC, posterior cingulate cortex; mPFC, medial prefrontal cortex; Ang, angular gyrus; PMFG, posterior middle frontal gyrus; IPL, inferior parietal lobule; Orb, orbital gyrus; MTG, middle temporal gyrus; AMFG, anterior middle frontal gyrus; Ins, insula; dACC, dorsal attention cingulate cortex; Sup, supplementary gyrus


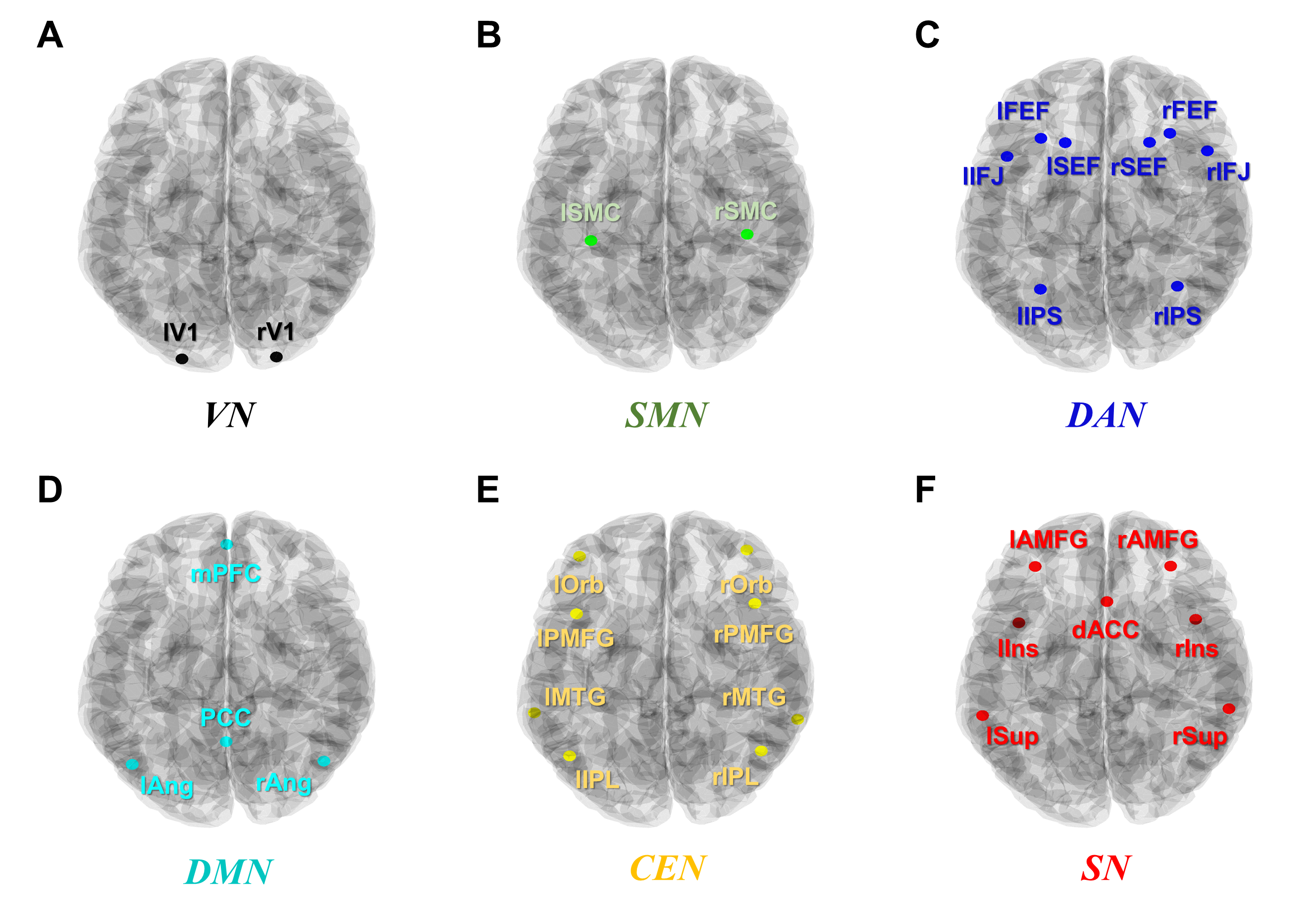


**Figure S1**. Positions of the ROIs mapped onto the brain template. Each panel corresponds to the module. The template image was acquired from the Brainstorm toolbox. (A) visual network, (B) somatosensory network, (C) dorsal attention network, (D) default mode network, (E) central executive network, and (F) salience network.

1.4. Determination of threshold for network binarization

The proportional thresholding approach allows for a comparison of network topologies under consistent network density, as the thresholding is applied using individual proportions. The individual proportional approach is known to be more stable than the absolute one [10-11]. In addition, recent studies have commonly reported that the multiple thresholding approach helps the network characteristics not to be biased [12-14].

To determine the optimal proportional threshold interval, a cluster-based permutation test was employed [15]. This statistical model identifies significantly different data clusters with mitigating criteria for the type 1 error. Specifically, the gCCs were compared between trMDD and grMDD.

**2. Supplementary results**

2.1. Group comparison for the numbers of epochs

There was no group difference in the number of epochs used in the analyses (Table S2).

**Table S2.** A comparison of the number of epochs used for the analyses. There was no significant group difference for the number of epochs.

| trMDD | grMDD | HC | *p* |
| --- | --- | --- | --- |
| 93.67 ± 29.52 | 99.09 ± 28.13 | 93.53 ± 30.04 | 0.1074 |

trMDD, treatment-refractory MDD; grMDD, good-responding MDD; HC, healthy control

2.2. Optimal proportional threshold interval

The cluster-based permutation test revealed a decreased gCC in trMDD ranging from 5 to 35% compared to grMDD (*p* = 0.0161, Figure S2). Furthermore, the network isolation (i.e., infinite characteristic path length) occurred in less than 5% up to a 35% of sparsity level (Figure S3), suggesting that excessive thresholding could destroy the small-world network properties. Consequently, the optimal threshold to differentiate trMDD from grMDD was established as the 5 to 35% range, which is in line with the previous finding [16]. Our data-driven thresholding approach might yield a more optimal threshold range to differentiate trMDD.


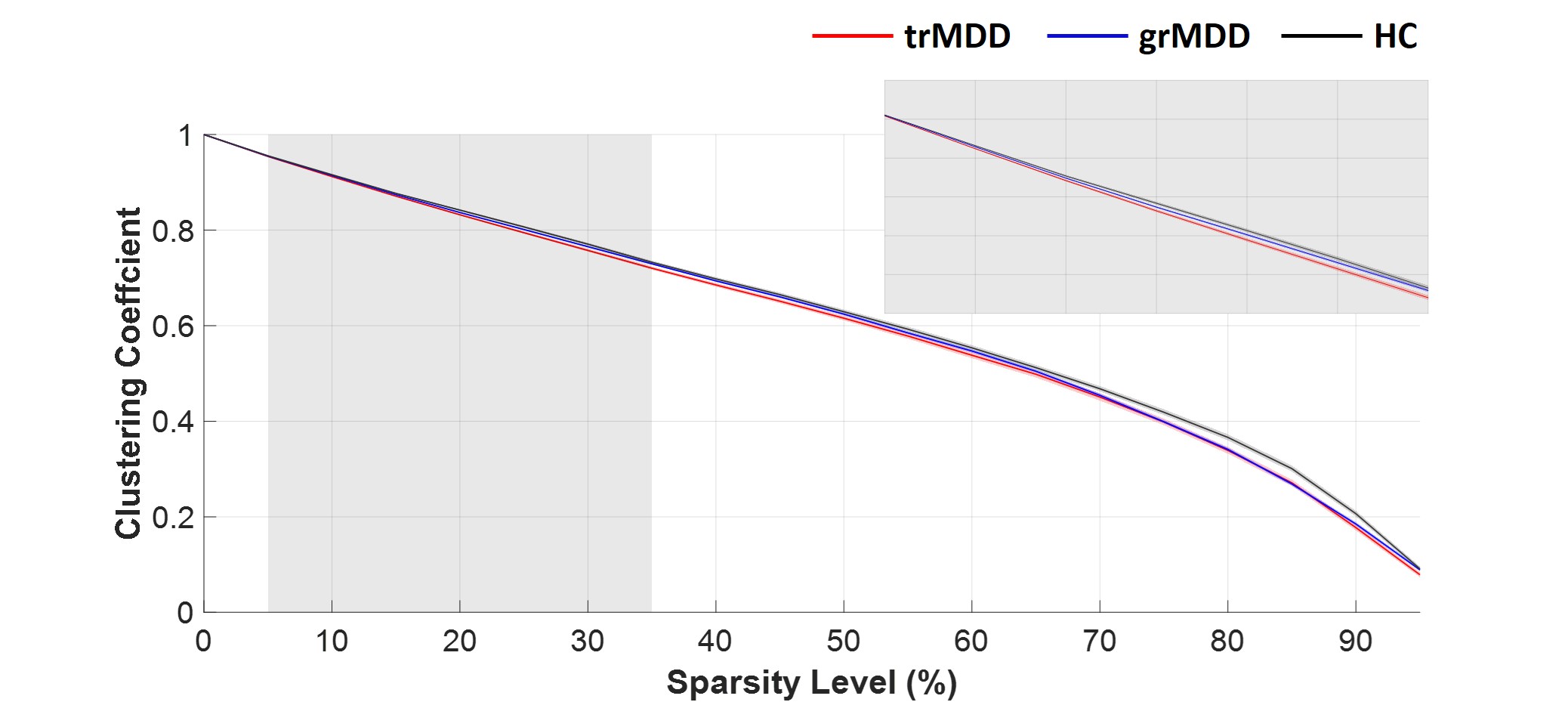


**Figure S2**. Global CC for each group according to the sparsity level. The gray box highlights the range within which trMDD showed significantly lower CCs compared to grMDD, as detailed in the magnified inset at the upper right. The red, blue, and black lines represent the grand-averaged global CC values for trMDD, grMDD, and HC, respectively, with the standard errors depicted as shaded areas around the lines.

trMDD, treatment-refractory MDD; grMDD, good-responding MDD; HC, healthy control; CC, clustering coefficient


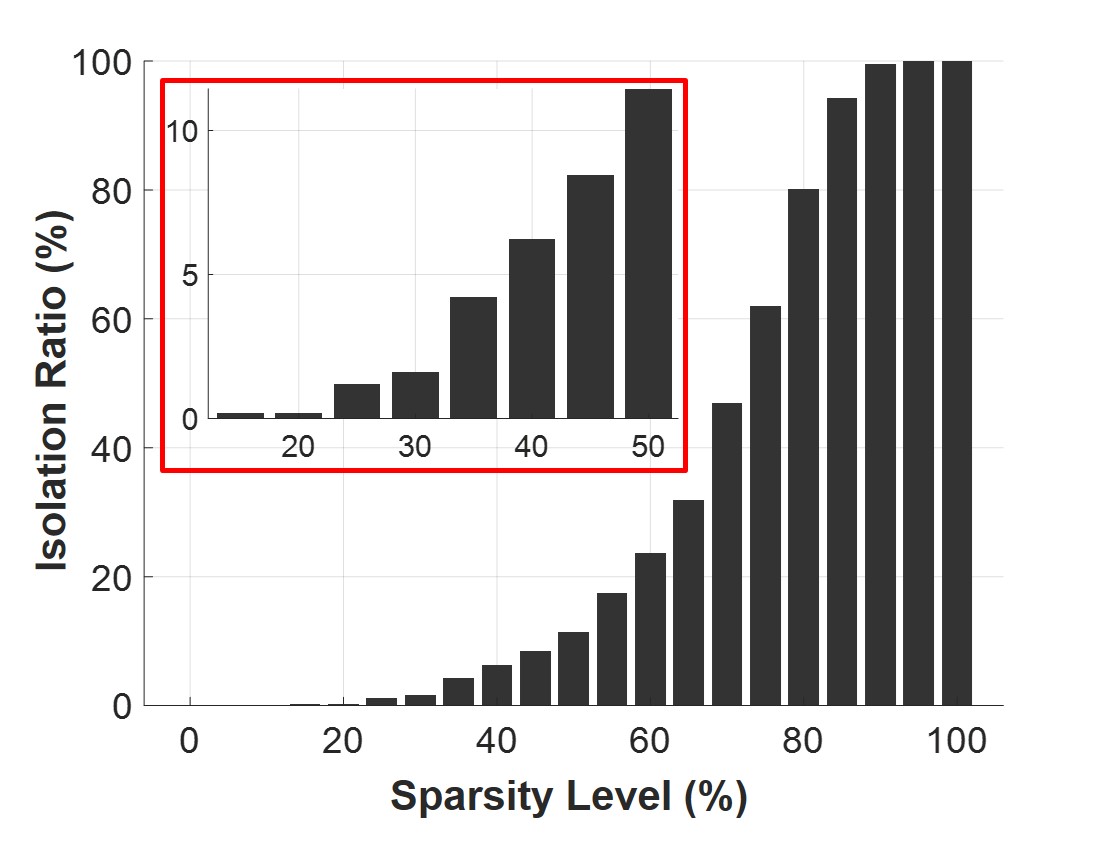


**Figure S3**. The ratio of the network isolation according to the sparsity level. The network isolation is defined as the cases where the network has infinite characteristic path length. The upper left inset is magnified to show the isolation ratio across sparsity levels ranging from 15% (the first non-zero value) to 50% (more than 10% of isolation). Notably, the isolation ratio remains below 5% for sparsity levels up to 35%.

2.3. NBS results


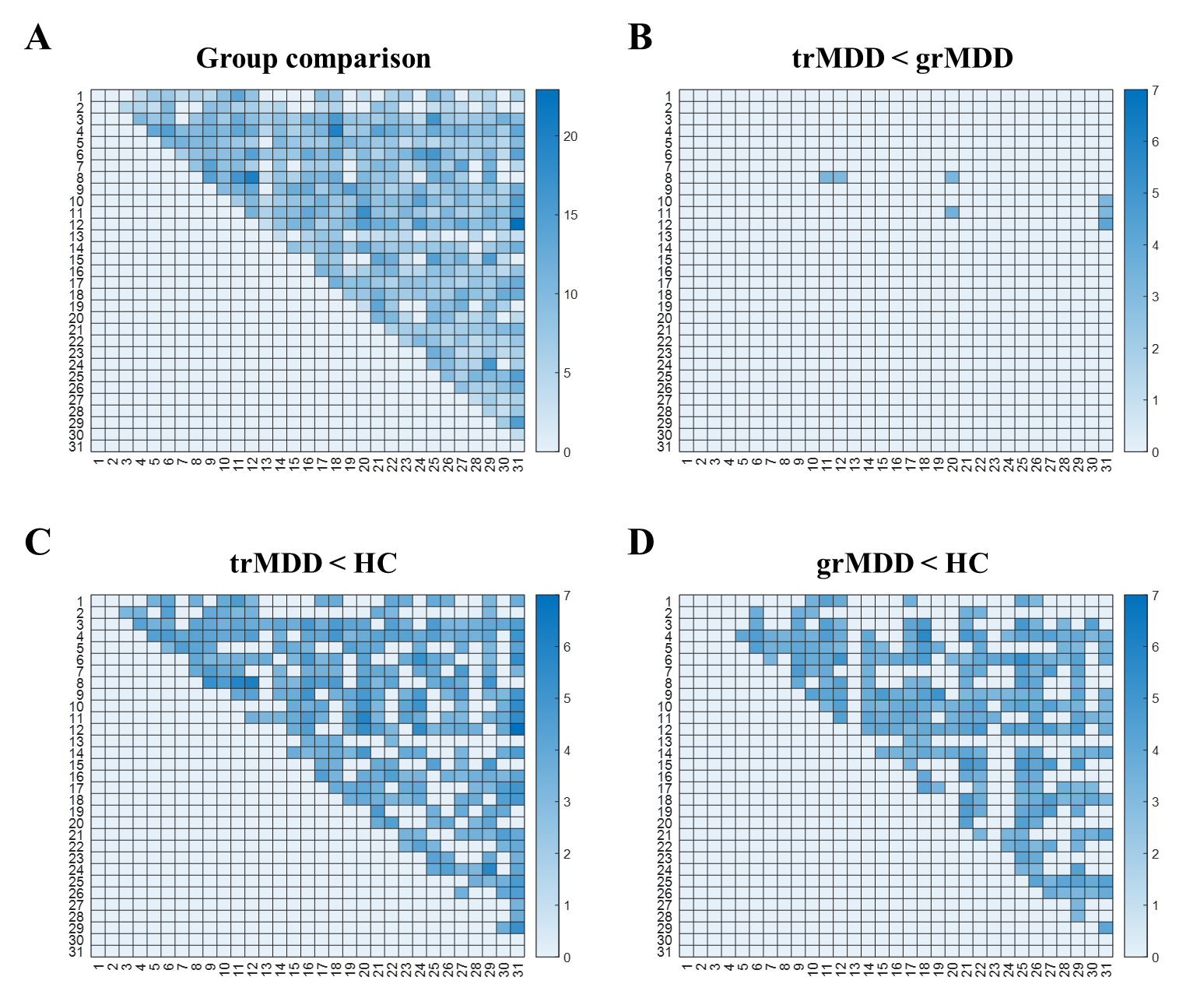


**Figure S4**. Comparative analysis results from the NBS. Only the regions showing significant group differences are displayed. The indices of the nodes correspond to the ROI indices, which are presented in Table S1. The values of the elements are transformed p-values through the application of the negative logarithm function (i.e., - log (.)). Only upper triangles are presented. (A) Results of the ANOVA model. (B) A post-hoc t-test between trMDD and grMDD. (C) A post-hoc t-test between trMDD and HC. (D) A post-hoc t-test between grMDD and HC. Additional post-hoc analyses were performed for the identified subnetwork in which trMDD and grMDD differ, using classic t-tests and FDR corrections.

2.4. Efficiency

2.4.1. Global efficiency (gEff)

As mentioned in the main article, the gEff values were almost similar. Specifically, 400 of 498 participants showed the same gEff values as 0.9002 (Figure S5). The same values are mainly due to the proportional thresholding method, which preserves FC density. Considering that the gEff is calculated as the inverse shortest path length, the same numbers of FCs in the binary networks could render the gEff values of them quite similar.


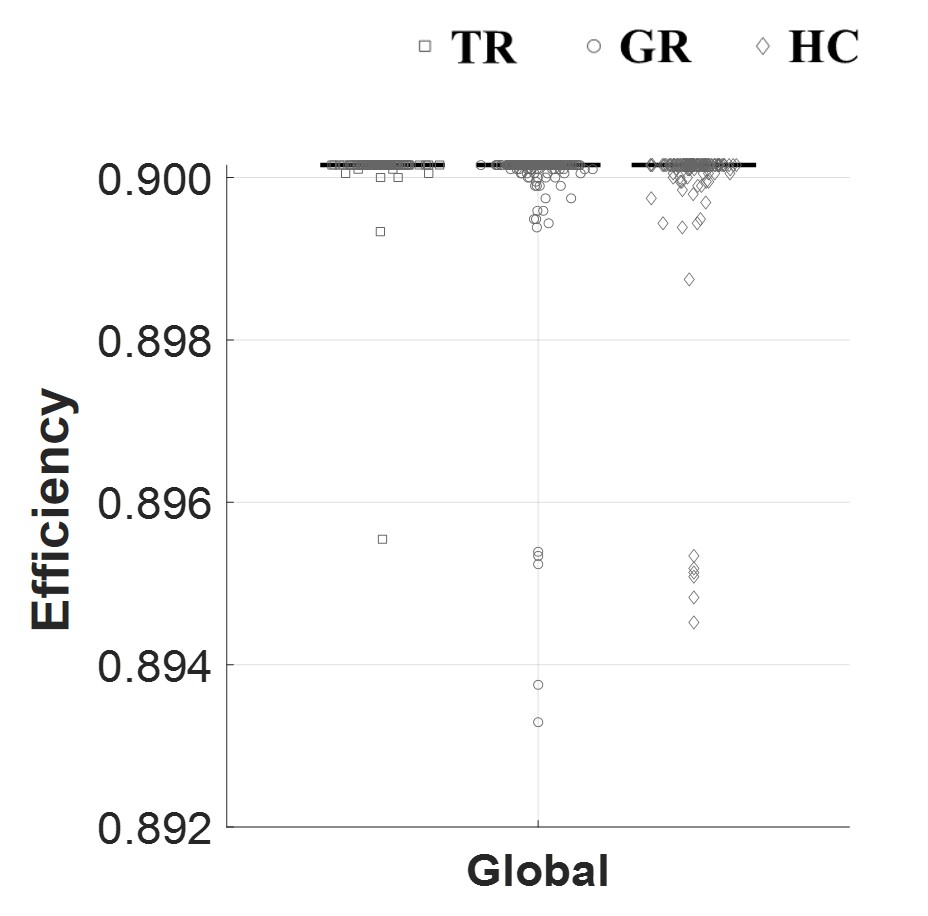


**Figure S5**. Group comparison for gEff. For each gEff, trMDD (square), grMDD (circle), and HC (diamond) data are sequentially presented. Each dot represents the individual gEff value, with their median values indicated with the central solid lines. It is noted that 400 samples showed same gEff values, 0.9002.

2.4.2. Nodal efficiency (nEff)

The Pearson correlation coefficients were calculated between all the nEff and nodal CC values for all nodes (i.e., 31 nodes) across all participants (i.e., 498). The nEffs showed a highly positive correlation with the nodal CCs (*r* = 0.9308, p < 0.0001; Figure S6).


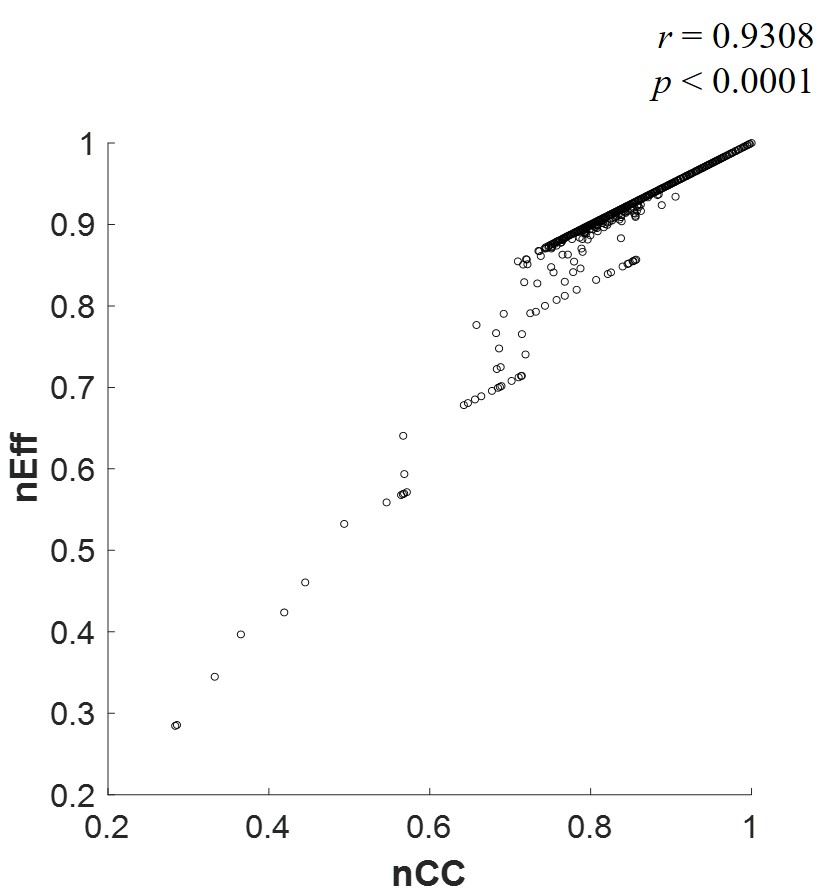


**Figure S6**. Correlation between the nodal clustering coefficients (nCC) and the nodal efficiency (nEff). In the scatter plot, all the nodal network measures (i.e., 31) from all participants (i.e., 498) are displayed. It is noted that nEff values are strongly dependent on the nCC values.

2.5. Network indices for the random networks (simulation)

From the randomly connected networks, nodal CCs, nEffs, and DCs were calculated and presented (Table S3). In the simulation, weighted symmetric matrices were randomly constructed 5000 times. The number of their nodes was set at 31. For each edge between nodes, a pseudo-random value was assigned from a uniform distribution ranging from 0 to 1. The network indices were computed from the binarized matrices, with the sparsity level across 5 to 35%. The random sampling was conducted using the ‘rand’ function implemented in MATLAB (MathWorks).

**Table S3**. The network indices calculated from the randomly constructed networks.

| Index | DC | CC | nEff |
| --- | --- | --- | --- |
| Values | 24.01 | 0.8003 | 0.9001 |

DC, degree centrality; CC, clustering coefficient; nEff, nodal efficiency

**References**

[1] Kroenke, K., Spitzer, R. L., & Williams, J. B. W. (2001). The PHQ-9: Validity of a brief depression severity measure. Journal of General Internal Medicine, 16(9), 606-613. https://doi.org/10.1046/j.1525-1497.2001.016009606.

[2] Choi, K. M., Kim, J. Y., Kim, Y. W., Han, J. W., Im, C. H., & Lee, S. H. (2021). Comparative analysis of default mode networks in major psychiatric disorders using resting-state EEG. *Scientific reports*, 11(1), 22007.

[3] Tadel, F., Baillet, S., Mosher, J. C., Pantazis, D., & Leahy, R. M. (2011). Brainstorm: a user-friendly application for MEG/EEG analysis. *Computational intelligence and neuroscience*, 2011, 1-13.

[4] Gramfort, A., Papadopoulo, T., Olivi, E., & Clerc, M. (2010). OpenMEEG: opensource software for quasistatic bioelectromagnetics. *Biomedical engineering online*, 9, 1-20.

[5] Chen, A. C., Oathes, D. J., Chang, C., Bradley, T., Zhou, Z. W., Williams, L. M., ... & Etkin, A. (2013). Causal interactions between fronto-parietal central executive and default-mode networks in humans. *Proceedings of the National Academy of Sciences*, 110(49), 19944-19949.

[6] Rolle, C. E., Fonzo, G. A., Wu, W., Toll, R., Jha, M. K., Cooper, C., ... & Etkin, A. (2020). Cortical connectivity moderators of antidepressant vs placebo treatment response in major depressive disorder: secondary analysis of a randomized clinical trial. *JAMA psychiatry*, 77(4), 397-408.

[7] Hipp, J. F., Hawellek, D. J., Corbetta, M., Siegel, M., & Engel, A. K. (2012). Large-scale cortical correlation structure of spontaneous oscillatory activity. *Nature neuroscience*, 15(6), 884-890.

[8] Toll, R. T., Wu, W., Naparstek, S., Zhang, Y., Narayan, M., Patenaude, B., ... & Etkin, A. (2020). An electroencephalography connectomic profile of posttraumatic stress disorder. *American Journal of Psychiatry*, 177(3), 233-243.

[9] Rubinov, M., & Sporns, O. (2010). Complex network measures of brain connectivity: uses and interpretations. *Neuroimage*, 52(3), 1059-1069.

[10] Hatlestad-Hall, C., Bruña, R., Syvertsen, M. R., Erichsen, A., Andersson, V., Vecchio, F., ... & Haraldsen, I. H. (2021). Source-level EEG and graph theory reveal widespread functional network alterations in focal epilepsy. *Clinical Neurophysiology*, 132(7), 1663-1676.

[11] Garrison, K. A., Scheinost, D., Finn, E. S., Shen, X., & Constable, R. T. (2015). The (in) stability of functional brain network measures across thresholds. *Neuroimage*, 118, 651-661.

[12] Bassett, D. S., Nelson, B. G., Mueller, B. A., Camchong, J., & Lim, K. O. (2012). Altered resting state complexity in schizophrenia. *Neuroimage*, 59(3), 2196-2207.

[13] Zhang, M., Zhou, H., Liu, L., Feng, L., Yang, J., Wang, G., & Zhong, N. (2018). Randomized EEG functional brain networks in major depressive disorders with greater resilience and lower rich-club coefficient. *Clinical Neurophysiology*, 129(4), 743-758.

[14] Adamovich, T., Zakharov, I., Tabueva, A., & Malykh, S. (2022). The thresholding problem and variability in the EEG graph network parameters. *Scientific Reports*, 12(1), 18659.

[15] Maris, E., & Oostenveld, R. (2007). Nonparametric statistical testing of EEG-and MEG-data. *Journal of neuroscience methods*, 164(1), 177-190.

[16] Li, G., Li, B., Wang, G., Zhang, J., & Wang, J. (2017). A new method for human mental fatigue detection with several EEG channels. Journal of Medical and Biological Engineering, 37, 240-247.
